# Becoming a valued member of society: the meaning of Art-Based vocational rehabilitation in the Norwegian labour and welfare service

**DOI:** 10.1186/s40359-025-02597-0

**Published:** 2025-03-18

**Authors:** Kristin Berre Ørjasæter, Dina von Heimburg

**Affiliations:** 1 Faculty of Nursing and Health Science, Division of Health and Community Participation, Nord University, Namsos, 7801 Norway; 2 Nordic Research Center for Wellbeing and Social Sustainability, Department of Education and Lifelong Learning, NTNU, Postboks, Torgarden, Trondheim, 8900, 7491 Norway

**Keywords:** Citizenship, Lifeworld research, Mattering, Phenomenology, NEET, Young people

## Abstract

**Background:**

Individuals with social, mental health and/or addiction issues often face significant barriers to accessing, maintaining, and re-entering education or employment. Exclusion from these domains is linked to feelings of marginalization, hopelessness, and a reduced sense of significance.

**Methods:**

In our study, following the phenomenological reflexive lifeworld research approach, we conducted 11 interviews with young people facing social, mental health and/or addiction issues, who have experienced being out of school and work. The aim was to explore the potential of art-based vocational rehabilitation (ABVR) from the perspective of young people.

**Results:**

The essential meaning of ABVR can be understood as a starting point on a journey for young people with complex issues in becoming valued members of society. The essential meaning is further explicated through its five constituents: [1] experiencing a sense of belonging [2], building friendship [3], empowering through goal-oriented rehabilitation [4], developing authenticity, and [5] believing that one matters.

**Conclusions:**

Participation in ABVR reshaped young people’s self-perceptions, their confidence in their abilities, and their sense of significance to others. Despite once living on the borders of society, they joined a community of like-minded peers, contributing value and feeling that they matter. Altogether, attending ABVR supported a sense of citizenship and mattering.

**Supplementary Information:**

The online version contains supplementary material available at 10.1186/s40359-025-02597-0.

## Introduction

This study explores the potential of art-based vocational rehabilitation (ABVR) to enhance education and employment for young people with social, mental health and addiction issues. Research has identified a link between young people’s mental health and substance use issues and their lack of engagement in education, employment or vocational training [1, 2]. In Norway, this concern is particularly acute, as young people not in education, employment, or training (NEETs) exhibit higher rates of psychosocial and health issues compared to their European counterparts [3, 4]. Norwegian NEETs are six times more likely to experience depression and four times more likely to report low life satisfaction than EU NEETs, who themselves face double the risk in both categories compared to the general youth population. These findings suggest that mental health issues are critical factors, reciprocally connected to NEET status [4].

To address this issue and promote educational attainment and workforce participation [5], targeted policies emphasizing early and intensive follow-up are crucial [6, 7]. However, Bond et al. [8] note that evidence-based vocational programs specifically targeting young populations remain scarce. Their evaluation of Individual Placement and Support (IPS), originally designed for adults with severe mental illness, demonstrated improved employment outcomes but limited impact on educational attainment. Similarly, a 2024 review by Stea et al. [9] found mixed results in interventions targeting NEETs, with five of nine studies reporting positive employment outcomes and three showing effects on education or training. Secondary benefits included improved mental health, self-esteem, and self-efficacy, as well as reduced health complaints and substance use. Young people with social, mental health, and addiction issues present unique challenges in assessing work, education, and vocational training [10–12]. This underscores the importance of initiatives like the “Youth Guarantee”, which advocates personalized and intensive support to increase success rates for NEETs. By fostering effective follow-up, it aims to reduce risks of social exclusion, hopelessness, and dependence on public benefits [13].

For young people, the Norwegian Labour and Welfare Administration (NAV) provides forms of social and economic security while encouraging transitions to activity and employment [14, 15]. NAV’s vocational rehabilitation services include tailored activities designed to strengthen individuals’ competencies and work readiness, thereby alleviating the adverse effects of unemployment [14]. Among these services, some employ ABVR to assist young people in overcoming barriers to education and employment.

Evidence indicates that art-based activities enhance health, wellbeing [16] and support mental health recovery [17, 18]. These activities are employed in various settings, including schools, community centers, hospitals, juvenile justice system, for purposes such as substance use prevention [19], relapse prevention [20], fostering mindfulness [21], building resilience [22], behavioral change [23] and promoting social and mental wellbeing [24, 25]. Key outcomes of art-based activities among young people include increased sense of belonging, resilience, knowledge, and ability to cope with difficult emotions and challenging situations [19, 22, 26, 27]. According to Golden et al. [28], the implementation of art-based activities for young people with complex issues offers an effective, accessible, and adaptable solution. However, some research questions the critical role of art-based activities in promoting health and reducing social and health inequalities, calling for more critical investigation into the topic [29].

Research on the labor market outcomes of art-based activities among young people are limited. However, Calero et al. [30] state that such activities can be an effective alternative to traditional pedagogical approaches to youth vocational training. Furthermore, Bruun et al. [31] found that participating in art-based activities influenced unemployed people’s confidence, motivation, and ability to obtain and maintain work and education. Nevertheless, given the lack of additional research, knowledge about the power of such activities in vocational rehabilitation remains slim.

Art-based vocational rehabilitation offers a valuable avenue for building networks of support. By participating in collaborative activities, young people can establish connections through shared creative experiences, with the arts serving as a “common third” that fosters mutual engagement and understanding. According to Fyhn et al. [32], relational challenges and a lack of belonging are among the most significant risk factors for NEETs in Norway. Addressing these relational deficits is critical, as supportive relationships in everyday life—including those with professionals possessing strong relational competencies—constitute essential protective factors. These relationships are pivotal in enabling young people to re-engage with education, training, or employment. The importance of these protective factors aligns with the theoretical frameworks underpinning this study. Drawing from theories on mattering [33–36] and citizenship [37, 38] the research explores how fostering a sense of belonging and agency can empower young individuals. These frameworks are briefly outlined in the following sections.

In this study, we build from Prilleltensky’s definition of mattering as an ideal state of complementary, highly interdependent psychological experiences: being valued and adding value—to oneself, others, work, and the broader community and society [36]. Being valued refers to feeling recognized, accepted, appreciated, and seen by others in ways that foster a sense of belonging and identity, while providing individuals with the assurance of their importance within relationships, organizations, and society. Adding value, on the other hand, involves making meaningful contributions that benefit oneself, others, organizations, and society at large. This entails engaging in purposeful actions that promote personal growth, support others, and address larger societal needs, thereby reinforcing self-worth and fostering a purpose-driven life [33]. If activities, relationships, and societies focus solely on individualistic experiences of “being valued,” they risk fostering “I-cultures” characterized by competition and narcissism. Thus, the need to feel valued must be balanced with the equally fundamental need to add value, aligning with the creation of “we-cultures” founded on reciprocal support.

While individuals may feel connected to groups and communities, such as through arts-based activities, they may not always perceive their contributions as valued or their dignity as upheld. Achieving a balance between being valued and adding value requires consistent attention to *conditions* that nurture a sense of mattering [34]. These conditions are deeply connected to social determinants of health and the structural and relational root causes that shape experiences of mattering and overall well-being. Prilleltensky [34, 36] underscores the importance of enhancing agency and empowerment, particularly for those most affected by social and work-life exclusion, enabling them to actively influence the factors that shape their lives.

Accordingly, experiences of mattering represent a necessary progression from mere feelings of belonging to active and equitable citizenship [34–36]. This concept emphasize the importance of empowerment, agency, and meaningful participation, thereby facilitating social change through inclusive social engagement [39, 40]. To complement theories of mattering, we use Rowe’s [38] citizenship framework, which identifies five core elements—rights, responsibilities, roles, resources, and relationships—that collectively foster social inclusion and participation. According to Ponce and Rowe [37], it is the responsibility of social services and society to support the full citizenship of individuals with disabilities by ensuring access to these elements, including education and employment opportunities.

Given the scarcity of effective employment services for young people [8], this study examines the experiences of young individuals with social, mental health, and addiction issues who have been out of school and work. It explores the potential of art-based activities in facilitating their access, maintenance, and return to work and education. This research provides new insights into the vocational and non-vocational impacts of these activities from the youths’ perspective, which has been scarcely explored before. The research question addressed is: *What are the experiences of young people with social*,* mental health*,* and addiction issues participating in arts-based vocational rehabilitation*,* and how do these activities facilitate their access to*,* maintenance of*,* and return to work and education?*

## Method

To explore how individuals live through, experience, act out, and describe an everyday phenomenon, we adopted Dahlberg et al.’s [41] phenomenological reflexive lifeworld research (RLR) approach. This approach assumes that uncovering ABVR’s potential requires an open engagement with the lifeworld of those who have experienced the phenomenon in question. Such openness, termed a “bridled attitude” [41], involves being present and maintaining awareness of the evolving understanding of the phenomenon and its multiple meanings.

### Setting

As part of a research project addressing ABVR for young adults in Norway, we examined two ABVR programs for young adults in NAV. Both programs are group-based and target young, unemployed people struggling with challenges due to mental health conditions, social and/or addiction disorders, isolation, disrupted circadian rhythms, and/or lack of mastery, as well as with finding meaning or motivation in life. Overall, the programs aim to identify young people’s interest and skills and thereafter facilitate their transitions to work, education, or training.

Program I is a group-based music program led by a musician employed by NAV. The program is offered in a community building with space for rehearsals and concerts; the program’s group uses the building in the daytime, while the building is open to the community during evenings and weekends. Participating in the program involves [1] producing music (e.g., creating music, writing lyrics, recording music, and being part of the house band; and [2] promoting events and concerts at the community building and at other venues in the city and surrounding area. The program operates Monday through Thursday from 09:00 to 15:00.

By contrast, Program II, now defunct, was a group-based music and media program financed by NAV but run by a private provider. The program’s staff included a coach, a musician, and a sound, light, and media technician. In 2020, the program ended after 8 years despite its high rate of success in placing young people in work, education, and training. During its years of operation, the program was offered at several locations, each with its own creative space. Participants largely performed self-produced works and engaged in different kinds of creative productions in the education, health, and/or cultural sectors at the local, regional, and national levels. The program operated every weekday from 09:00 to 15:00.

### Data collection

The data for this study derived from a research project on ABVR for young people in Norway. Two contact persons, or *gatekeepers*, were established, one at each NAV office. They were responsible for recruiting participants based on the inclusion criteria: [1] participants had to be between 16 and 35 years old, and [2] currently participating or having previously participated in an art-based vocational program aimed at young people who are out of school or work. To ensure rich data variation, we also asked gatekeepers to invite participants who differed in age, gender, roles, and program attendance duration, and satisfaction.

Since the sample size in RLR could not be pinpointed before the examined project was underway, we began our study with eight participants and were prepared to include additional participants as the study continued. Due to the complexity of the phenomenon and the need for rich data variation, we expanded the sample to 11 young people, aged 21 to 31. Variation in data is crucial in reflective lifeworld research to provide a comprehensive understanding of the phenomenon. Saturation is viewed not as the absence of new information, but as achieving a rich, varied understanding that captures the depth and complexity of lived experiences [41].

Information sheets detailing the study’s nature, purpose, and participation requirements were provided digitally to the gatekeepers, who then forwarded them to prospective participants in person, over the phone, and/or via email. Those interested in the study either contacted the first author directly or allowed the gatekeeper to share their contact information so we could provide more details, and they could decide whether to participate in an interview.

To collect data, we conducted semi structured interviews. The interviews were conducted in a conversational format and followed the principles of an open RLR approach with the aim of understanding the meaning of a phenomenon in everyday life. All interviews were conducted by the first author and began with the question, “Would you please describe the art-based program that you attend(ed)) at?” The participants were encouraged to speak freely and to describe their experiences in as much detail as possible. During the interviews, the first author posed additional questions, including “Would you please tell me when and how you have been/were affiliated with the art-based program?”; “Would you describe your role and tasks in the program?”; and “What experiences have you had/did you have with the program?” For an even deeper understanding, follow-up questions were also posed, including “Would you please give an example?”; “Would you elaborate on that?”; and “Would you please describe what you mean by that?”

Data collection proceeded from August 2021 to August 2023. Most interviews were conducted in person at the location of the art-based program or at the university where the first author is employed; all the other interviews were conducted virtually. The interviews ranged from 30 to 75 min in length, were audio-recorded, and were later transcribed verbatim.

### Data analysis

In analyzing the data, we adopted an attitude of alertness, allowing the phenomenon to reveal itself using the RLR approach [41]. The researcher acted as a “hunter of meanings”, delving below the surface to seek a deeper understanding beyond initial appearances. This approach involved observing what was given rather than hastily determining its meaning. To achieve our goal of “making definite what is indefinite,” we had to “dwell” with the phenomenon as long as possible [41].

Reflexivity was integral to ensuring the validity and reliability of our findings. This involved continuous self-awareness and critical reflection by the researcher. We employed “bridling”, which entails refraining from pre-understanding and setting aside personal beliefs, theories, and assumptions that could limit openness [41]. To maintain reflexivity throughout the process, preliminary findings were discussed with various stakeholders, which was crucial as the initial analysis was conducted solely by the first author.

In the first step of analysis, the first author read and reread the interview transcripts multiple times to grasp each as a whole. This step was deeply reflective, with the author continuously noting down reflections and questions that arose during the reading. These annotations were central when organizing the data into smaller parts, “meaning units”, keeping them as close as possible to the original statements. The first author maintained self-awareness by continuously questioning how personal biases could influence the organization of data, particularly regarding mental health recovery. This reflexive approach was integral to the analysis, ensuring that the interpretations remained grounded in the participants’ original statements. The meaning units were then organized into clusters and named [41]. To enhance understanding and reflexivity, the first author presented and discussed the findings with various stakeholders, including individuals out of school and work, NAV staff and managers, health- and social workers, and researchers at meetings, workshops and conferences. The first author encouraged stakeholders to share their diverse perspectives, ensuring a comprehensive and reflective analysis. At that time, four thematic groupings of meaning, or clusters, were presented: [1] social network; [2] belonging; [3] authenticity; and [4] positive experiences. Based upon their feedback and discussion, the first author revisited the data and refined the analysis. Throughout this iterative process, the first author engaged in discussions with the second author and the research group in mental health, with which the first author is affiliated. These discussions were crucial for challenging and refining the emerging essence and its interpretation. By actively listening to and incorporating the insights and critiques from co-author and researchers in the mental health research group, the first author was able to deepen the understanding and enhance the robustness of the analysis. During this process, it became clear that the meaning of having concrete goals to work towards and believing that one matters were not sufficiently highlighted in the data, necessitating a greater emphasis on these aspects. Finally, the clusters were organized into patterns that collectively generated a structure of meaning, representing the essence (i.e., core meaning) of the ABVR and its constituent parts: [1] experiencing a sense of belonging [2], building friendship; [3] empowering through goal-oriented rehabilitation; [4] developing authenticity, and [5] believing that one matters. In this final step, the second author and the research group in mental health were actively involved, challenging the essence and its interpretation, and discussing its theoretical and research implications.

### Ethical considerations

Our study was assessed by the Centre for Research Data (No.783042). A pre-assessment request for the study was also submitted to the Committee for Medical and Health Research. Because the committee (No. 309516) considered the study to not be a medical or health-related project, it fell outside the Health Research Act and was thus not subject to further ethical approval.

## Findings

In its essential meaning, ABVR can be described as a journey toward full membership in society. Entering ABVR can transform young people’s understanding of who they are, what they can achieve, and what significance they have for others. The young people who participated in ABVR became part of a community of peers with different experiences, competencies, and aims in life who created memorable experiences together. They were provided with access to a medium that made it possible to identify, understand, and express their own feelings. They also came to believe in their personal value by virtue of their being human and their ability to contribute to their own lives and the lives of others.

That essential meaning was broken down into five constituent parts: [1] experiencing a sense of belonging [2], building friendship [3], empowering through goal-oriented rehabilitation [4], developing authenticity, and [5] believing that one matters. Those constituent parts represent different aspects of the phenomenon but are not mutually exclusive. On the contrary, they somewhat overlap and together form the phenomenon as a whole.

### Experiencing a sense of belonging

Young people with social, mental health, and addiction issues who are not in employment, training, or education can experience existence outside their communities. In characterizing their everyday lives, participants described being isolated at home and feeling alone in the world. However, their comments highlighted the importance of being included in a community. In ABVR, they could meet in person and be part of a group with like-minded people, even during the COVID-19 pandemic.

As Ibrahim described it,

When you have nothing to do in your everyday life, as in my case—I just sat in my room and played computer games—then you’re locked in with yourself, doing nothing more than looking at a PC screen, day in and day out. Then you become trapped. In ABVR, I became part of a group. (Ibrahim)

For those spending most of their time alone, ABVR became a long-awaited social meeting place and a support in creating a routine.

You get a place to go where you can do things. Doing something is great when you’re at home almost all of the time. I’m a routine-oriented person, so it’s nice to come two days a week to sing and play with a whole bunch of people. (Bea)

The fact that ABVR targets young people with various issues helped to create a sense of belonging: “It may not be so nice to say that it’s fine that everyone’s struggling with something. However, when you struggle, it’s nicer to be together with other people who are having the same experience” (Kaira).

A “we culture” arose by participating in ABVR. The participants reported experiencing that they no longer felt alone with their problems. Instead, they became a relationally tight-knit group that supported and helped each other and, as a result, became stronger together:

Most of us were far from the normal life. We were there together and tried figuring out what to do together. We could scream together, we could yell at each other, but we could also agree. We were almost like siblings. Indeed, the respect that arose became essential. (Milla)

They thus gained a sense of inclusion and became part of a community, or “a herd.” The people in ABVR became their herd in a community where they felt a sense of belonging.

### Building friendships

ABVR created social bonds between young people. As Ailo put it, “The strength of [ABVR] is socialization.” Before participants entered ABVR, their social contact with peers was limited. Several expressed missing interactions with people their own age but were simultaneously unsure whether they knew the social codes in youth environments or had developed sufficient social skills to know how to behave in different situations that could arise:

Coming here, being able to be with someone and doing things together, makes a big difference when you haven’t experienced that otherwise. Being social, being with someone, expanded my bubble to be able to be more receptive to people and new things. I’ve formed greater social bonds as a result. (Bea)

Although some participants already had social networks that they expanded by getting to know new people, others reported that friendship was virtually absent in their lives before they attended ABVR: “I went from having almost no friends when I came here to having many good friends” (Bea).

Young people who had previously been bullied by peers could experience a feeling of being insecure in social gatherings. In ABVR, participants were given the time and security to have positive experiences interacting with peers. In that light, ABVR became an important arena to practice socially interacting with peers and forging close ties with individuals their own age:

[Being in ABVR] has helped a lot. What I feel most is that it’s much easier now than when I started here to talk to people, to socialize, and to associate with people my own age. I struggle a lot with anxiety, and the reason why is that I was bullied very severely.… I’m very insecure around people my own age. And, as a result, I haven’t had many friends. So, it’s helped a lot already. Like today, for example, I’ve been sitting and chatting all day with different people. (Frida)

However, this was not something that came immediately or naturally from day one; some participants needed a long time to experience it.

By contrast, one participant had never struggled in social settings and had a significant social network. Upon witnessing how much the other participants had struggled; he decided to make his own network available to them. That gesture cost him little but had a significant impact for his peers:

I’ve always had a lot of friends, always been social. So I’m in a position to make a positive contribution to others. They really appreciate it, because it provides them with other opportunities. (Gabriel)

However, for the vast majority of participants, ABVR became a place where they could form social bonds. As a result, some participants established new networks, while others expanded theirs with others in the program.

### Empowering through goal-oriented rehabilitation

Young people with social, mental health, and addiction issues can struggle with a lack of motivation in life, particularly if they have neither identified nor set concrete goals that they could work toward. Without having future plans and dreams, participants struggled to have a healthy bedtime routine. There were few glimmers of light in their lives, and the meaning they found waking up in the morning was limited. This was also the case for Gabriel when he started ABVR. At first, he was usually late, meaning that the house band had to start rehearsal without him. A conversation with the staff member about his future plans and dreams became a turning point: “I was told that if I wanted to be recommended for a job, then I’d have to arrive [for rehearsal] on time for at least a month straight, to show that I can manage it.” The staff member told Gabriel that he saw his outstanding professional potential but that punctual attendance would be crucial if he wanted to succeed on the labor market. If Gabriel wanted a recommendation from the staff to make a living in the light and sound industry, then he would have to improve his morning and bedtime routines. With the staff’s recommendation as an incentive to work toward achieving a normal circadian rhythm, Gabriel at last found motivation to get up in the morning:

For me, it was just a matter of starting to take responsibility for myself.… It went rather quickly and took only a couple of weeks before the routine [morning and bedtime routine] was established. It’s interesting what you can achieve when you suddenly have the motivation to get up. (Gabriel)

ABVR provided participants with opportunities and memorable experiences to overcome personal issues, break boundaries, and build confidence, ultimately fostering both personal and professional growth. For instance, Liam’s experience of overcoming performance anxiety gave him affirmation and the belief that he was capable of breaking boundaries in life in general. Lian shared:

I had performance anxiety that was out of this world. [The music staff] told me to drop it and face the situation, to just go ahead and play, plain and simple. I ended up playing in the big concert hall; it was quite fantastic. I managed to break boundaries, get on stage, and play in front of people. I get paid to do that today. In my full-time job, I have work tasks that differ dramatically from what I’ve done before, but you know, I can break boundaries, and I truly believe that I can manage it. He [the music staff member] was absolutely right: it worked. I have taken away the life lesson that it’s [performing on stage] not as dangerous as it looks at first glance; you just have to try, and when you take the chance, you’ll most likely succeed. (Liam)

Some participants expressed concerns about a goal-oriented structure, fearing it might increase their stress and exacerbate their experiences of failure. Originally, ABVR was intended to be a short-term program focused on employment, education and vocational training. However, in practice, many participants remained in the program for extended periods, with the primary aim being to lead meaningful lives, where employment or education was just one component. Frida, for instance, resisted the imposition of specific milestones within a set timeframe, stating:

My goal is simply to improve, to function better. Completing my education, getting a job, and pursuing further education are my objectives. However, setting rigid deadlines only causes stress and leads to failure if things don’t go as planned. I know NAV operates this way, but I do not. (Frida)

Overall, goal-oriented rehabilitation empowers participants by helping them establish healthy routines, build confidence, and achieve personal and professional growth, even if some initially resist structured goals due to stress.

### Developing authenticity

Among the participants, it was quite common to feel insecure or be afraid of not fitting in. They were used to playing roles that they assumed were expected of them in order to be accepted or for others to tolerate them. In ABVR, however, they gained experience that they could fully be themselves, that there was room to show the less favorable aspects of themselves, and that there was more to life than sunny days:

You fit in the way you are; it’s delicious.… Although I’m sometimes in bad shape, I can come here. I can sit down and have a cup of coffee. That’s okay. I can do the work tasks at my own pace. (Celine)

Bea made this visible by telling her story about not needing to wear a “mask” in ABVR. She learned that she did not need to suppress aspects of herself that might differentiate her from other participants:

I no longer feel stressed about being different. When you have problems like I do, you disguise yourself and put a veil in front of who you really are, so that nobody thinks that you’re weird. Here, I’ve learned that I don’t have to mask myself. It’s been quite important [to learn that]. Because I’ve usually felt that showing more of myself is too much for others to deal with to the point that they don’t want to hang out with me. (Bea)

The participants welcomed finding a way to express how they feel inside. As several reported challenges identifying and expressing their emotional lives in words, ABVR provided participants with a space where they could dare to start expressing how they really feel inside. Milla, one of the young participants, described her attendance in ABVR as a turning point in her ability to express her feelings. For her, music became important for identifying with, understanding, and processing emotions in life that she had no language for:

It’s so hard to put things into words, but music makes it possible. So, in my worst bouts of depression, I only listened to songs about how bad life was.… Today, I don’t need to do that. Now, I know myself and have no need to use music to understand myself. I have the language to talk about it, to express myself. (Milla)

In ABVR, the participants were given the opportunity of using their experiences and emotions to create lyrics that could inform people around them about how they feel without having to talk about it:

It’s nice to express my emotions through singing, because they are uncomfortable to show or express in conversation.… If I’ve had a triggering day, then singing helps me. It’s easier to express my emotions in lyrics than to talk about them. (Bea)

By participating in ABVR, participants increasingly became more honest with themselves and others about their personal situations. When Frida began training for work, she strove to be open and experienced that doing so created greater understanding and empathy from the people around her:

I’ve learned to be more open about it [my situation]. In my current job, I’ve been very open, told them [coworkers] what kind of challenges I struggle with, like somatic pain, depression, and anxiety. They know that days with lot of pain can be hard for me and that I might not be able to show up. Their response has been, “We can adjust for that.” That’s been very helpful. I don’t need to destroy myself by pretending that I’m fine. Instead, I can say “Yes, today is a pretty hard day. But I’m here. I don’t know if I can stay for the whole day, but I really want to try.” Then, they respond, “You stay as long as you manage it.” (Bea).

Not everyone felt comfortable sharing their struggles. Ailo describes:

I rarely talk about my issues with others. I might say I’m tired and lie down during rehearsals, claiming I forgot to take my Ritalin. In reality, I burn out both physically and mentally. I always give more than I can handle, and after a few months, it becomes overwhelming. I usually end up needing to sleep and stay in bed, struggling to get up and participate. I’m afraid it will happen again, as it has from high school to folk high school, but I have no strategies to manage it and can’t share it with other participants or NAV staff (Ailo).

Participants were afforded a space where they could break down some of the walls that they had built during life. Most experienced that it was safe to be genuine and that embracing themselves and their emotions was possible without compromising oneself. The arts thus became quite important in their process of becoming authentic.

### Believing that one matters

Most participants had long been out of work, education, and training and felt as though they were more of a burden than a resource. In ABVR, however, they began to believe that they matter. They gained the opportunity to learn something new but also to teach others. For example, David, who had completed higher education in music, gained experience with making a contribution by sharing his musical expertise:

The others in the group are very good and have lots of talent, but they don’t have a background in music theory as I do. Therefore, I’ve become a quasi-educator in the group.… I only play guitar [not his primary instrument], because it provides a greater opportunity to show more technical aspects, to explain how chords work and how they are built up. (David)

Some participants indeed had limited experience with music but could share experiences and skills related to other areas of life. That dynamic fostered equality and made it visible that everyone could contribute something to the group:

I had limited experience [with music], so I’ve learned a lot. It was a little nerve-wracking when everyone had more musical knowledge than me. At the same time, think about how much we can learn from each other! During the years I’ve been here, I’ve learned a lot, so it’s really been perfect being a novice, particularly when the others are eager to teach me.… For my part, I have relational skills. They refer to me a lot when new people arrive in the group, because I’m pretty good at spotting people and their needs. (Celine)

ABVR offered participants experiences that underscored their capacity to make a difference and that their contributions were appreciated and beneficial not only to themselves but also to others: value for others, not just themselves:

The feeling here is that people need you. I’ve noticed that when someone’s away from the group, it quickly falls apart. So you kind of don’t want to put the others in that situation.… Some days, I can’t manage to come because it’s too difficult to walk out the door at home. When others later say that those days were particularly bad for the group, it’s good to hear. (David)

In ABVR, participants were able to create something beautiful based on difficult lived situations and emotions. That ability was viewed as being not only useful for themselves but also important for others who are struggling:

I write about what I struggle with mentally. In that sense, writing is a way to process it.… It’s better to turn it into a song than to talk about it. That makes it a little bit easier. So, I write lyrics to get things out of my body. By getting it down on paper, getting it formulated, I create something. Then, it’s no longer just something negative. It’s something new and occasionally something beautiful as well. (Frida)

On the whole, ABVR supported the participants by helping them go from feeling inadequate or useless to feeling that they had valuable traits and could help others despite having problems in their own lives.

## Discussion

Our findings indicate that ABVR can serve as a starting point for individuals on their journey to becoming valued members of society. In this section, we discuss the essential meaning of ABVR within the conceptual framework of citizenship and mattering.

Our findings indicate that young people in ABVR initially felt disconnected from mainstream society due to isolation at home, limited social networks, and avoidance of the education system and labor market. Consequently, their perceived degree of citizenship was low. According to Pettersen [42], citizenship can be categorized into three classes; full, second-class, and non-citizen. The young people in our study were at risk of second-class citizenship due to their marginalization in society. Despite their willingness, their civil rights, including social inclusion and participation, were not adequately protected. For individuals facing complex issues, striving for equal rights equates to striving for full membership in society [37, 42]. Ponce and Rowe [37], identify two paths to achieving status as a full member of society: [1] putting forth individual effort and receiving support with gaining access to full citizenship and [2] asserting society’s responsibility to create access to citizenship.

Similar to other vocational programs, ABVR aimed to enhance individuals’ knowledge, skills, and opportunities for educational or employment success, thereby reducing reliance on sickness, unemployment, and disability benefits [15]. However, ABVR did not solely focus on personal initiative and effort to access the workforce. It also emphasized society’s responsibility to provide young people with relevant knowledge and skills for the labor market. This includes offering necessary opportunities and resources for those with social, mental health and addiction issues to manage work, study or training, thereby facilitating access to citizenship. The young people gained access to resources, support, and tools to understand themselves, life, and the working world. Together, this support strengthened their access to the 5Rs and their sense of belonging in society. Consistent with Rowe [38], our findings illustrate the importance of addressing more than just transitions to work, education, and training. Emphasizing the emotional needs of young people, including forming friendship, fostering a sense of belonging, and living authentically, all of which are central to empowering agency and achieving full citizenship. In that light, participating in vocational rehabilitation can contribute to “recovering citizenship” [43].

Our findings also show that the young people in ABVR gained a feeling of social worth and being important to a group. These experiences can be understood as what Prilleltensky [36] has referred to as *mattering -* that one is valued and can add value in their own lives and in the wider society. The young people in our study were provided with experiences in which they could gain mastery as well as resources and competences that others appreciate. Although they all had vulnerable, challenging lives, they experienced themselves as being significant and as important sources of support for each other [44]. According to Prilleltensky [36], the experience of realizing one’s value has great importance in one’s life, whereas a perceived lack of significance tend to accelerate marginalization and illness. Because arts-based activities in the vocational rehabilitation programs were group-based, the young people had to work together, and thereby, develop soft skills and creative capabilities. As a result, they gained experience with depending on each other in order to produce and deliver high-quality musical productions and to develop empowerment as citizens. Moreover, their peers’ statements that rehearsals fell apart when they were not present made them realize their valuable contribution to the group. In line with the conceptual framework of mattering, the participants created a sense of worth; they felt valued by others, and they added value to the wider community. When they gained the feeling of mattering and sensed that they had social worth, their experiences could be transferred to other areas of life and to their relationships with society at large. Prilleltensky & Prillenltensky [34] has underscored that the more that individuals feel valued, the greater their confidence that they have value to add. That dynamic seemed true in our study as well. When the young people felt valued, they were able to add value to themselves, the group, and society.

The importance of adding value to the self may be at the heart of how people come to experience being a full member of society [45]. The work that each young person performed to embrace themselves and start the process of unmasking themselves seemed especially important. Without any need to hide aspects of themselves, they realized that they could in fact add value and become a valued member of society.

Because the young people in our study had not found a group to belong to for years, they were social beings with a fundamental need to belong [46] and evolutionarily programmed to seek a “we culture” [34]. In ABVR, they were provided access to a community, a “we culture,” that provides a source of friendship, reciprocity and active citizenship. In line with Nord-Baade & Rowe [45], the young people learned that they were tolerable despite their limitations, while recognizing the warmth and appreciation from peers and staff made it possible for them to balance adding value to others and adding value to themselves. As Prilleltensky [36] has underscored, “To feel worthy, we have to feel that we are equal to others and that we deserve to be treated with respect.”

Traditionally, vocational rehabilitation has used the “train then place” approach, involving stepwise employment preparation through unpaid work, apprenticeships, or sheltered training. Recently, there has been a shift towards “place then train” approach, which secures competitive employment based on participants’ goals and provides ongoing support after employment is obtained [47]. Research in Norway indicates that the traditional approach often creates a parallel society rather than genuine inclusion and belonging, and can increase the distance to employment and education [48–50]. The ABVR programs in this study can be seen as either “train then place”, using art-based activities as a training arena without job placements outside NAV, or as an “in-between” approach due to their numerous performances. Surprisingly, participation in ABVR seemed to increase the likelihood, motivation, and confidence to secure employment or resume education. It also enhanced non-vocational outcomes, such as strengthening friendships, fostering a sense of belonging, authenticity, and feeling valued. This aligns with Stea et al. [9], who found that vocational programs can improve secondary outcomes related to self-perception and life situation.

Given that participants in this study remained in ABVR for 1–2 years, it is reasonable to consider whether IPS or other vocational program might be more effective. It is important to note that some participants had previously attempted work placement in companies, both with and without staff support, without success. This could be due to factors such as timing, feeling unprepared, or lacking necessary skills. The success in ABVR may be attributed to the ability to begin training at a lower level with high flexibility. Similar to Sabella [51], the flexibility and supportive environment, including the facility, staff, and peers, were crucial. Another factor could be the art aspect of the vocational rehabilitation program. As Varkøy [52] suggests, the arts provide an opportunity to reflect upon being in the world. The young people found that playing music together involved more than just learning skills; it provided a space for self-understanding, social interaction, and societal connection. The art-based group-oriented element likely contributed to their sense of reconnection.

### Strengths and limitations

Due to challenges with recruiting participants and conducting interviews amid COVID-19 and sick leave, recruitment for our study lasts 2 years. However, that lengthy recruitment period afforded us an unique opportunity to adopt a reflective attitude and remain flexible in understanding the phenomenon under study in new, manifold ways instead of merely confirming what is already known [41]. In short, because we did not seek a “quick fix” understanding, the meaning of ABVR was allowed to mature.

The data represented both current and previous participants in ABVR, which can be viewed as both a limitation and a strength. Whereas past participants had to speak about ABVR in retrospect and were liable to forget nuances, current participants could have been too close to the phenomenon and without the necessary distance to perceive what meaning ABVR might have in their lives. At the same time, the variation in the data provided us with more nuances of the phenomenon.

This phenomenological study aimed to develop insight into participation in art-based vocational rehabilitation from the experiences of young people. Its strength lies in the rich and detailed descriptions of the phenomenon, revealed through the lived experiences of the participants. The findings are highly context-dependent, deeply rooted in the specific experiences and context of the study. In line with phenomenological approaches, we seek to broaden the understanding of the phenomenon rather than explain or generalize findings. While the study captured the potential of art-based vocational rehabilitation, our descriptions allow other researchers to use our findings in planning future research and determining applicability to similar contexts and groups. However, to generalize findings to a larger population, future research should incorporate standardized metrics or validated questionnaires to measure outcomes such as quality of life or skill improvements. This approach could provide a more comprehensive understanding of the outcomes, achievable through a mixed-methods approach combining phenomenological insights with quantitative data.

Last, our data mainly represented participants who were confident in ABVR, as we struggled to recruit those who were dissatisfied or had dropped out. This difficulty may stem from using NAV employees familiar with ABVR, but not directly involved in the programs, for recruitment. Despite our efforts to include a diverse sample, the participants generally had a positive view of ABVR. However, we included interview questions about the program’s weakness and suggestions for future improvements to ensure a comprehensive evaluation.

## Conclusion

Participating in ABVR allowed young participants to realize that their current circumstances could change, offering them steps towards a different everyday life. In ABVR, they engaged with all eight parts of the mattering wheel—not all equally but enough to describe their transitioning from isolation and limited meaningful activities to developing friendship, gaining recognition, and experiencing mastery. This shift moved them from marginalized existence to a more nuanced and fulfilling life, where they felt valued and could contribute to themselves, the group, and society. The question remains whether these outcomes are specific to ABVR or if they could be achieved through other vocational rehabilitation programs. It may not be the art itself that is decisive, but rather the program’s design, which enables staff and peers to recognize and acknowledge individuals’ potential and identity, fostering a sense of belonging and mattering.

### Practical implications

When designing vocational rehabilitation programs for young people, it is important to consider the concepts of mattering and citizenship. Our study indicates that young people who have disengaged from work and education require more than just training and skills for accessing and maintaining employment and education. They often lack opportunities to form friendships and experiences that foster mastery and self-efficacy. Therefore, it is crucial to create environments where they can develop competencies beyond formal competence (i.e. credits), interact with peers, and feel safe to express their authentic selves. Art-based activities can act as a vital stepping stone toward work and education, providing participants with essential initial steps in the right direction and instilling hope for the future. By fostering environments where individuals can both feel valued and contribute meaningfully, systems and organizations can empower individuals to achieve well-being and participate in societal change. For example, educational settings, workplaces, and community programs that integrate practices of recognition and opportunities for meaningful contribution though arts-based activities and groups, can enhance individuals’ sense of mattering [34]. In such settings, they can learn to face challenges while feeling significant to themselves and others. Developing a more nuanced understanding of their abilities and potential is essential. For individuals with complex issues to achieve full citizenship, they need to feel a sense of belonging, significance, and value [34, 37, 45]. This recognition of personhood can occur in interactions with staff [53], and peers. Additionally, providing spaces where they can interact with like-minded individuals can benefit both the individuals and society. To achieve this, society must assume greater responsibility for individuals and not place excessive responsibility on individuals or any single service.

Our qualitative phenomenological study reveals interesting findings on art-based vocational rehabilitation, including reshaping self-perception, enhancing confidence, and fostering a sense of citizenship and mattering. These findings should be considered alongside other studies and evidence. As it is premature to draw definitive conclusion from a single study, we suggest expanding vocational rehabilitation programs and simultaneously researching their impact on young people’s transition to work, education, and training, as well as their emotional needs. Investigating how these programs are utilized and perceived by young people can guide future recommendations for integrating them into broader employment and educational policies, highlighting the significance of feeling valued and attaining full citizenship.

## Electronic supplementary material

Below is the link to the electronic supplementary material.

Supplementary Material 1

## Data Availability

No datasets were generated or analysed during the current study.

## Abbreviations

ABVR: Art-based vocational rehabilitation
NAV: The Norwegian Labour and Welfare Service
RLR: Reflexive Lifeworld Research
