# Supplementary material for: Becoming a valued member of society: the meaning of Art-Based vocational rehabilitation in the Norwegian labour and welfare service
Source: BMC Psychol. 2025 Mar 18;13:269. doi: 10.1186/s40359-025-02597-0 (PMC11921480; doi:10.1186/s40359-025-02597-0)
Supplement: Supplementary file 1 — Supplementary Material 1 [file 40359_2025_2597_MOESM1_ESM.docx]

**REACTIVATE: Interview guide**

The interview guide will be used as a preparation and guide for the interviews. It consists of themes and questions that the researcher will explore in order to answer the overall research questions. The interviews are planned to be flexible and conversational, rather than strictly structured.

**Introduction before the start of the interview:**

The researcher leading the interview will initially introduce herself and describe how the collected data material will be used and presented. In addition, information will be given briefly about the interview, the participants’ rights (research ethics)and the researcher's responsibility for confidentiality and anonymisation of data material.

**Young participants**

- Tell me about yourself and your background?
- How would you describe/define an art-based program?
- Would you please tell me when and how you have been/were affiliated with the art-based program?
- Which art-based programs have you been involved in?
- What does it mean to participate in this/these program(s)?
- Why did you want to participate in an art-based program?
- Would you describe your role and tasks in the program?
- How did you work artistically in the program?
- What did you want to achieve by participating in this program? Please describe
- What experiences have you had/did you have with the program?
- What has participating in art-based program(s) meant for you and/or your life?
- How do you describe the strengths and weaknesses of the program(s)?
- What do you experience as needed to make such programs helpful?
- Is there something you would like to elaborate on in terms of art-based programs and your participation in such a program?

**Employees**

- Tell me about your professional background and work experience?
- How would you describe/define an art-based program?
- Would you please tell me when and why you started working in an arts-based program?
- Which art-based program(s) have you been involved in?
- What is/was the aim of offering an art-based program?
- Who is/was the target group?
- What philosophy and working methods characterized the program?
- Please describe how you worked with young people in the program?
- Would you describe your role and tasks in the program?
- What meaning provide(d) such program in young people and in their life?
- What strengths and weaknesses have you identified with the program?
- What is needed to make art-based programs helpful for young people?
- What barriers do/did you experience running such a program?
- If you were given the opportunity to develop a new art-based program, how should it look like and why?
- Is there anything you would like to elaborate on in terms of art-based programs and your contribution in such programs?
